# Case Report: Post-surgical Guillain-Barré syndrome as a rare differential diagnosis of flaccid paralysis of the lower extremities in an infant after cardiac surgery

**DOI:** 10.3389/fped.2025.1610035

**Published:** 2025-06-06

**Authors:** Maximilian Gross, Rafal Berger, Felix Neunhoeffer, Johannes Nordmeyer, Andrea Bevot

**Affiliations:** ^1^ Department of Pediatric Cardiology, Pulmonology and Pediatric Intensive Care Medicine, University Children’s Hospital Tübingen, Tübingen, Germany; ^2^ Department of Thoracic and Cardiovascular Surgery, University Hospital Tübingen, Tübingen, Germany; ^3^ Department of Neuropediatrics, General Pediatrics, Diabetology, Endocrinology and Social Pediatrics, University Children’s Hospital Tübingen, Tübingen, Germany

**Keywords:** pediatric Guillain-Barré syndrome, post-surgical flaccid paralysis, coarctation, case report, cytomegalovirus

## Abstract

**Introduction:**

Guillain-Barré syndrome (GBS) is an important cause of flaccid paralysis in children and is mainly associated with antecedent infections. Surgery as an additional trigger for GBS is a well-documented phenomenon in adults, but is significantly less reported in pediatric patients. This case report describes an infant with post-surgical GBS following cardiac surgery, highlighting the diagnostic challenges and differential diagnoses of post-surgical GBS in the pediatric intensive care setting.

**Case description:**

A former extremely preterm infant with congenital cytomegalovirus (CMV) infection underwent a second surgery for re-coarctation of the aorta with aortic arch hypoplasia at the chronological age of six months. While requiring extracorporeal membrane oxygenation postoperatively, the girl presented with flaccid paralysis of the lower extremities. Magnetic resonance imaging of the brain, spine, and nerve conduction studies demonstrated findings consistent with acute motor-sensory axonal neuropathy-type GBS. She was treated with intravenous immune globulin and ganciclovir due to CMV reactivation (plasma 14,000 copies/ml). Gradual neurological improvement was noted over the following months, while persistent motor deficits remained, suggesting potential disease transition into chronic inflammatory demyelinating polyneuropathy.

**Conclusions:**

This case report emphasizes the importance of considering post-surgical GBS in critically ill children with postoperative paralysis. Recognition may be delayed due to variable initial presentations and accompanying factors such as sedation and extracorporeal life support.

## Introduction

Guillain-Barré syndrome (GBS) is one of the most common causes of acquired flaccid paralysis in children, with an incidence of 0.62–0.75 cases per 100,000 person-years (1, 2). GBS is extremely rare in infants, peaking at preschool age and mid-adolescence, and affects males more often (1, 3).

Although not fully understood, GBS is thought to result from an immune-mediated process in which autoantibodies targeting epitopes on peripheral nerves lead to demyelination and axonal loss (4). In consequence, a rapidly progressive polyneuropathy with sensory symptoms, ascending symmetric muscle weakness, cranial nerve involvement, and autonomic dysfunction may occur, often accompanied by neuropathic pain (2, 4). Several subtypes of GBS have been described based on clinical and neurophysiological findings, including acute inflammatory demyelinating polyneuropathy, acute motor axonal neuropathy, and acute motor-sensory axonal neuropathy (AMSAN) (2, 5, 6).

Regarding triggering factors for GBS, antecedent bacterial or viral infections of the respiratory or gastrointestinal tract are thought to play a significant role, as infections are reported in most childhood and adult GBS cases (3, 7). In adults, other triggers, such as certain medications, bone marrow transplantation, trauma, and surgery have been linked to GBS (8). Post-surgical GBS has been reported following various surgeries, including heart surgery, with widely varying incidences of 4.5 up to 51.5 per 100,000 surgeries (9, 10). Compared with that in adults, post-surgical GBS in children is either significantly less common or more often undiagnosed, as only a few case reports exist, with no mention of surgery-associated GBS in children in either large cohorts or literature reviews (1, 9, 11–13).

We report a case of a former extremely preterm infant with coarctation of the aorta (CoA) and congenital cytomegalovirus (CMV) who presented with GBS after cardiac surgery. On postoperative day three after patch aortoplasty of the aortic arch for re-coarctation, the girl presented with bilateral flaccid paralysis of the lower extremities with absent reflexes. Potential autonomic dysfunction included arterial hypertension and paralytic ileus. Cranial and spinal magnetic resonance imaging (MRI) showed a strong contrast enhancement of the dura at the level of the lumbar spine, including the spinal nerve roots and the cauda fibers. Repeated nerve conduction studies indicated leg-focused polyradiculoneuropathy with peripheral axonal neuropathy without other demyelinating characteristics. Lumbar puncture revealed normal cell counts with slightly elevated cerebrospinal fluid (CSF) protein within the normal range relative to the girl's age. While onconeural and antiganglioside autoantibodies were not present in the cerebrospinal fluid or the serum, CMV reactivation was detected in the plasma.

## Case description

The girl was delivered via cesarean section at a gestational age of 24 weeks and 6 days due to an abnormal Doppler ultrasound with intrauterine growth restriction at the Children's University Hospital Tübingen, Germany. At birth, her weight was 375 grams (z-score: −2.68), her length was 20 cm (z-score: −5.75), and her head circumference was 20 cm (z-score: −1.71) (14). A primary CMV infection was diagnosed during pregnancy. Postnatal treatment in the neonatal intensive care unit (NICU) included respiratory support via continuous positive airway pressure, intermittent mechanical ventilation, and surfactant administration. The initially suspected and later confirmed diagnosis of a congenital CMV infection was treated with ganciclovir and valganciclovir for 6 months, leading to undetectable CMV viral loads in the plasma and urine. Postnatally, the girl was diagnosed with CoA with aortic arch hypoplasia, patent foramen ovale, and several ventricular septal defects (VSDs) located in the perimembranous and muscular ventricular septum. During the NICU stay, prostaglandin infusion was used to maintain patency of the ductus arteriosus until surgical repair of the CoA. Concomitantly, milrinone, furosemide, propranolol, and captopril were administered as medically indicated. The girl was transferred from the NICU to the pediatric intensive care unit (PICU) for further treatment of the heart defect. During her NICU stay, she was diagnosed with severe bronchopulmonary dysplasia and stage 3 retinopathy of prematurity in addition to the abovementioned diagnoses. Apart from being small for gestational age, initial thrombocytopenia, and leukopenia, no typical sequelae associated with congenital CMV infection were observed during the NICU stay. Neurological examination, hearing evaluation by auditory brainstem response, and neuroimaging, including ultrasonography and cerebral MRI, were normal.

CoA repair with resection and extended end-end anastomosis, and ligation of the ductus arteriosus via left thoracotomy were performed without complications at the chronological age of three months. Owing to postoperative recurrent CoA, the girl underwent unsuccessful balloon angioplasty one month later. Therefore, at the chronological age of six months, the patient underwent a second operation involving patch aortoplasty of the aortic arch using bovine pericardium, closure of the VSDs, and closure of the patent foramen ovale. The cross-clamp time was 165 min. Postoperatively, severe myocardial dysfunction with particularly impaired right ventricular function required a brief course of cardiopulmonary resuscitation (CPR) and a switch from cardiopulmonary bypass to venoatrial extracorporeal membrane oxygenation (VA-ECMO) before the girl was re-admitted to the PICU. PICU management included treatment with inhaled nitric oxide, iloprost, sildenafil, bosentan, milrinone, levosimendan, hydrocortisone, catecholamines, and deep sedation and analgesia, as indicated. On the second postoperative day, VA-ECMO weaning failed, requiring 30 min of CPR and re-initiation of VA-ECMO. Subsequently, atrioseptectomy was performed on the sixth postoperative day, leading to successful VA-ECMO weaning two days later.

On the third postoperative day, the girl presented with bilateral flaccid paralysis of the lower extremities with absent reflexes. Potential autonomic dysfunction included arterial hypertension and paralytic ileus. Intracranial and intraspinal hemorrhaging were excluded by ultrasound. After VA-ECMO weaning, cranial and spinal MRI revealed no evidence of ischemia or hemorrhage but an increased gadolinium enhancement of the oculomotor nerves, the internal acoustic meatus on both sides, and a strong contrast enhancement of the dura at the level of the lumbar spine, including the spinal nerve roots and the cauda fibers. Repeated nerve conduction studies performed initially and two weeks later indicated leg-focused polyradiculoneuropathy with peripheral axonal neuropathy. Findings included markedly reduced distal motor amplitudes, absent F-waves, and preserved distal latencies without conduction block or temporal dispersion — consistent with AMSAN-type GBS (Table 1). Lumbar puncture revealed normal cell counts with slightly elevated cerebrospinal fluid (CSF) protein within the normal range relative to the girl's age (52 mg/dl) (15).

**Table 1:** Motor and sensory nerve conduction studies.

Motor nerve conduction study (reference values in parenthesis)			
	Initial studies	After 3 weeks	After 20 weeks
Left median nerve	DML: 1.5 ms (2.9 ms) Amp: 16.1/12.8 mV (0.9 mV) CV: 21 m/s (21 m/s) F wave: –	DML: 1.9 ms (2.9 ms) Amp: 4.6/2.8 mV (0.9 mV) CV: 34 m/s (21 m/s) F wave: –	
Right median nerve	DML: – Amp: – CV: – F wave: a wave only	DML: – Amp: – CV: – F wave: non-inducible	DML: 1.5 ms (2.2 ms) Amp: 17.4/17.9 mV (5.3 mV) CV: 26 m/s (44 m/s) F wave: 12.6 ms (≤18.7 ms)
Left tibial nerve	DML: 2.6 ms (3.2 ms) Amp: 0.7 mV (0.5 mV) CV: – F wave: non-inducible	DML: 1.8 ms (3.2 ms) Amp: 2.2/1.4 mV (0.5 mV) CV: 33 m/s (27 m/s) F wave: non-inducible	DML: 3.0 ms (2.7 ms) Amp: 0.4/0.3 mV (3.3 mV) CV: 28 m/s (48 ms) F wave: –
Right tibial nerve	DML: non-inducible Amp: non-inducible CV: non-inducible F wave: non-inducible	DML: 2.1 ms (3.2 ms) Amp: 1.1/0.8 mV (0.5 mV) CV: 33 m/s (27 m/s) F wave: non-inducible	DML: 2.1 ms (2.7 ms) Amp: 1.8/1.2 mV (3.3 mV) CV: 28 m/s (48 m/s) F wave: 21.4 ms (≤27.9 ms)
Sensory nerve conduction study (reference values in parenthesis)			
	Initial studies	After 3 weeks	After 20 weeks
Right median nerve	SL: 0.7 ms (3.2 m/s) Amp: 24.3 µV (5.5 µV) CV: 23 m/s (22 m/s) SNAP: –	SL: 1.2 ms (3.2 ms) Amp: 16.7 µV (5.5 µV) CV: 25 m/s (22 m/s) SNAP: –	
Left sural nerve		SL: non-inducible Amp: non-inducible CV: non-inducible SNAP: non-inducible	
Right sural nerve			SL:. Amp: obscured (artifact) CV: 45 m/s (44 m/s) SNAP: inducible

Table 1 shows the results of the motor and sensory nerve conduction studies. Amp., amplitude; CV, conduction velocity; DML, distal motor latency; SL, sensory latency; SNAP, sensory nerve action potential.

Onconeural and antiganglioside autoantibodies were not detected in the cerebrospinal fluid or the serum. Following the suspected diagnosis of Guillain-Barré syndrome, the girl was treated with two courses of intravenous immune globulin 400 mg/kg for five days each, one month apart. Owing to suspected neuropathic pain, treatment with gabapentin was initiated. While ruling out potential antecedent infections, CMV reactivation was detected, with a significantly increased CMV viral load in plasma (14,000 copies/ml) and urine (1,110,000 copies/ml) but not in CSF. Before the second surgery, no CMV was detectable in the blood or urine. The girl was subsequently treated with a second course of ganciclovir and valganciclovir for six weeks. In addition, extensive infectious disease workup ruled out HSV-1/2, Varicella-Zoster Virus, Epstein–Barr Virus, Human Herpesvirus-6, HIV-1/2, Hepatitis B and C, Measles, Mumps, Rubella, Tick-borne Encephalitis Virus, Entero-, Rota, Norovirus, Campylobacter spp., Salmonella spp., Shigella spp./Enteroinvasive E. coli, Yersinia enterocolitica, Clostridium difficile, Aeromonas spp., and Vibrio spp. Physical therapy was initiated and is ongoing while writing this manuscript.

Sixty-five days after the second surgery, the girl was transferred to another hospital closer to home, still requiring high-flow nasal cannula support, and was discharged home from that hospital.

Lower extremity motor function and nerve conduction studies revealed slight improvements in the months following the second surgery. The latter was performed five months after the second surgery and demonstrated severe axonal damage with evidence of proximal conduction recovery (Table 1). At the chronological age of 12 months (adjusted age of 9 months), the girl had a GBS Disability Scale score of 4/6 (16, 17). She exhibited paraplegia with preserved hip flexion, allowing leg elevation, whereas knee flexion and extension and ankle dorsiflexion and plantar flexion were absent bilaterally. Deep tendon reflexes were absent at the ankles bilaterally, whereas patellar reflexes were absent on the right and diminished on the left. The plantar response was absent bilaterally, with no plantar grasp reflex. Babinski's sign was negative on both sides. Sensory examination revealed no response to painful stimuli in either foot. On the Standardized Infant NeuroDevelopmental Assessment (SINDA) neurological and developmental scales, the girl scored 18 (maximum score 28) and 7 (maximum score 15) points, respectively, thus resulting in an atypical and slightly atypical score. These findings correspond with the above findings of neurological impairments and mild developmental delay. For the socio-emotional scale, a normal result was recorded (18).

## Discussion

Surgery is a known trigger for GBS in adults (8). As post-surgical GBS in children is much less common, this association is either much weaker or underdiagnosed (1). Kubert et al. described a case of a 13-year-old boy with severe autonomic dysfunction, seizures, and posterior reversible encephalopathy syndrome due to the acute motor sensory axonal neuropathy subtype of GBS. The first symptoms occurred on postoperative day seven. Gradual recovery was achieved with antihypertensives, pain management, and physiotherapy. The authors did not report on immune globulin treatment or plasmapheresis (12). Another case report of a 13-year-old boy described a severe disease progression of a preoperatively diagnosed GBS already treated with immune globulin therapy after abdominal surgery. Within hours after surgery, the boy required intubation and mechanical ventilation due to respiratory failure, with gradual recovery following a five-day course of 400 mg/kg per day of immune globulin and supportive care (11).

Owing to a history of aortic surgery and anticoagulation under VA-ECMO, we initially suspected that spinal cord injury or hematoma was the cause of the observed paraplegia. CoA repair generally has excellent outcomes, with low perioperative mortality rates usually <1% to 2% (19). The complications of CoA repair include re-coarctation, postoperative hypertension, pleural effusion, phrenic and left laryngeal nerve injury, and spinal injury (20–24). Spinal cord injury associated with CoA is rare and presents with postoperative lower extremity weakness or paralysis due to inadequate perfusion of the spinal cord. CoA-repair-linked spinal cord injury is believed to be multifactorial, with cross-clamp time, poor collateral circulation, and interruption of intercostal arteries being contributing factors (25, 26).

The initial diagnostic options were limited, as the girl was on VA-ECMO for seven days after surgery. Therefore, we ruled out major intracranial and spinal hemorrhage via ultrasonography. After VA-ECMO weaning, brain and spinal cord MRI showed no evidence of ischemia or hematoma, thus ruling out the initially suspected diagnosis of CoA repair-associated spinal injury. Instead, we detected gadolinium enhancement of the spinal nerve roots, which is often associated with GBS in children (27). Subsequent motor and sensory nerve conduction studies corroborated the MRI findings, leading to the initiation of immune globulin treatment for GBS. We opted for immune globulin treatment instead of plasmapheresis, as the latter has not been shown to be superior in pediatric patients and would not have been a feasible option in our case because of the girl's critical condition (28). Paresis was first detected on postoperative day three, within the range of manifestations of post-surgical GBS reported in the literature (9). However, a potential earlier occurrence could not be completely ruled out because deep analgesia and sedation were carried out up to that point. The latter could have also been why we could not observe a progression of paresis, as it had already reached its maximum at the time of diagnosis. Additionally, rapid-onset paresis is commonly observed in patients with the axonal subtypes of GBS, also indicated by our nerve conduction studies (9). We did not observe any upper extremity or cranial nerve involvement during the clinical examination. The respiratory muscles did not appear to have been significantly affected, as ventilatory weaning was achieved without complications after the end of VA-ECMO therapy. We observed arterial hypertension and paralytic ileus as potential manifestations of GBS-related autonomic dysfunction, although both are common phenomena without GBS in children after aortic surgery in the PICU (22).

We had to differentiate post-surgical GBS from critical illness polyneuropathy (CIP) and critical illness myopathy (CIM), which remains challenging in the intensive care unit setting (9, 10, 29). While our patient underwent a prolonged course of cardiopulmonary bypass, which is known to result in systemic inflammatory response syndrome and, therefore, is a trigger for CIP and CIM, other risk factors such as prolonged treatment with steroids and neuromuscular blocking agents were not present. Furthermore, our patient experienced flaccid paralysis limited to the lower extremities without generalized muscle weakness or difficulties in respirator weaning, which is usually observed in CIP and CIM (30). Additionally, our patient presented with severe sensory nerve involvement, autonomic dysfunction, and spinal nerve root gadolinium enhancement, the former being usually mild if present at all and the latter two being virtually non-existent in CIP and CIM (9, 31).

We found no metabolic or electrolyte disorders and refrained from testing vitamin levels (e.g., thiamine and B12) because the patient received partial parenteral nutrition with vitamin supplementation.

While investigating other possible triggers for GBS, we detected reactivation of CMV in the girl's peripheral blood. CMV reactivation is well described in critically ill patients (32, 33). Preceding CMV infection is a known factor associated with GBS in adults and children (7, 34), although, in the latter, the relationship between CMV infection and GBS is still debated (34, 35). In adults, GBS after CMV reactivation due to immunodepression after bone marrow and solid organ transplantation is rare and lacking in children, at least to our knowledge (36). As CMV reactivation could not ultimately be ruled out as a contributing factor, we therefore initiated ganciclovir treatment for 6 weeks as an individualized treatment attempt.

Given the prolonged clinical course and the absence of significant neurological recovery over several months, we hypothesize that the girl's GBS has transitioned into chronic inflammatory demyelinating polyneuropathy (CIDP), despite not fully satisfying the electrodiagnostic criteria with predominantly axonal involvement and no clear demyelinating features (3, 28, 37). Nevertheless, we evaluated glucocorticoid therapy as a subsequent treatment approach (28).

## Conclusion

Post-surgical GBS in children is rarely reported and involves several diagnostic pitfalls, especially when other causes are also possible for the observed signs and symptoms of polyneuropathy. Although uncommon, early consideration of post-surgical GBS as a potential cause of progressive muscle weakness with prompt treatment is crucial for improving patient outcomes.

## Data Availability

The datasets presented in this article are not readily available because the dataset contains sensitive and potentially identifiable patient information. In accordance with institutional and ethical regulations, the raw data cannot be shared publicly. However, relevant excerpts of the dataset (e.g., summary of nerve conduction studies, laboratory values, and clinical course) are described in the manuscript. Further details may be made available upon reasonable request and with appropriate institutional and ethical approvals. Requests to access the datasets should be directed to maximilian.gross@med.uni-tuebingen.de.
