# Supplementary material for: Case Report: Post-surgical Guillain-Barré syndrome as a rare differential diagnosis of flaccid paralysis of the lower extremities in an infant after cardiac surgery
Source: Front Pediatr. 2025 Jun 6;13:1610035. doi: 10.3389/fped.2025.1610035 (PMC12179211; doi:10.3389/fped.2025.1610035)
Supplement: Supplementary file 1 [file Table1.docx]

**CARE Checklist** for „Case report: Post-surgical Guillain-Barré syndrome as a rare differential diagnosis of flaccid paralysis of the lower extremities in an infant after cardiac surgery”

| Topic | Item | Checklist item description | Reported in Section |
| --- | --- | --- | --- |
| Title | 1 | The diagnosis or intervention of primary focus followed by the words “case report” | Title |
| Key Words | 2 | 2 to 5 key words that identify diagnoses or interventions in this case report, including "case report" | Key words |
| Abstract | 3a  3b  3c  3d | Introduction: What is unique about this case and what does it add to the scientific literature?  Main symptoms and/or important clinical findings  The main diagnoses, therapeutic interventions, and outcomes  Conclusion—What is the main “take-away” lesson(s) from this case? | Abstract |
| Introduction | 4 | One or two paragraphs summarizing why this case is unique | Introduction |
| Patient Information | 5a  5b  5c  5d | De-identified patient specific information  Primary concerns and symptoms of the patient  Medical, family, and psycho-social history including relevant genetic information  Relevant past interventions with outcomes | Introduction, Case description |
| Clinical Findings | 6 | Describe significant physical examination (PE) and important clinical findings. | Introduction, Case description |
| Timeline | 7 | Historical and current information from this episode of care organized as a timeline | n/a |
| Diagnostic Assessment | 8a  8b  8c  8d | Diagnostic testing (such as PE, laboratory testing, imaging, surveys)  Diagnostic challenges (such as access to testing, financial, or cultural)  Diagnosis (including other diagnoses considered)  Prognosis (such as staging in oncology) where applicable | Introduction, Case description |
| Therapeutic Intervention | 9a  9b  9c | Types of therapeutic intervention (such as pharmacologic, surgical, preventive, self-care)  Administration of therapeutic intervention (such as dosage, strength, duration)  Changes in therapeutic intervention (with rationale) | Introduction, Case description, Discussion |
| Follow-up and Outcomes | 10a  10b  10c  10d | Clinician and patient-assessed outcomes (if available)  Important follow-up diagnostic and other test results  Intervention adherence and tolerability (How was this assessed?)  Adverse and unanticipated events | Introduction, Case description, Discussion |
| Discussion | 11a  11b  11c  11d | A scientific discussion of the strengths AND limitations associated with this case report  Discussion of the relevant medical literature with references  The scientific rationale for any conclusions (including assessment of possible causes)  The primary “take-away” lessons of this case report (without references) in a one paragraph conclusion | Discussion |
| Patient Perspective | 12 | The patient should share their perspective in one to two paragraphs on the treatment(s) they received | n/a |
| Informed Consent | 13 | Did the patient give informed consent? Please provide if requested | Yes |
